# Effect of Melatonin on 24‐Hour Urinary Protein Excretion and Sleep Quality in Patients With Stage 3–4 Chronic Kidney Disease: A Randomized, Double‐Blind, Placebo‐Controlled Clinical Trial

**DOI:** 10.1002/hsr2.73458

**Published:** 2026-10-29

**Authors:** Shadi Ziaie, Behnam Mahdavi, Fatemeh Pourrezagholi, Aylar Nadiri Niri, Mohammad Abbasinazari, Roja Asadpour

**Affiliations:** ^1^ Department of Clinical Pharmacy, School of Pharmacy Shahid Beheshti University of Medical Sciences Tehran Tehran Province Iran; ^2^ Independent Researcher Saeed Pathology Lab Tehran Tehran Province Iran; ^3^ Chronic Kidney Disease Research Center (CKDRC), Department of Nephrology, Labbafinejad Hospital Shahid Beheshti University of Medical Sciences Tehran Tehran Province Iran; ^4^ Department of Cell and Molecular Biology University of Tehran Tehran Tehran Province Iran

**Keywords:** chronic kidney disease, melatonin, proteinuria, randomized controlled trial, renal function, sleep quality

## Abstract

**Background and Aims:**

Proteinuria is an established marker of renal injury and is closely associated with adverse outcomes in chronic kidney disease (CKD). Melatonin has attracted interest as a potential renoprotective agent because of its antioxidant, anti‐inflammatory, and regulatory effects on several pathways implicated in kidney injury. However, clinical data regarding its effects on proteinuria in patients with non‐dialysis CKD are limited. This trial investigated whether melatonin supplementation alters 24‐h urinary protein excretion in adults with stage 3–4 CKD.

**Methods:**

We conducted a 12‐week, randomized, double‐blind, placebo‐controlled clinical trial in adults with stage 3–4 CKD and persistent proteinuria. Seventy‐three participants were allocated in a 1:1 ratio to receive either oral melatonin (3 mg at bedtime) or a matching placebo. The prespecified primary endpoint was the change in 24‐h urinary protein excretion between baseline and week 12. Secondary assessments included renal function, blood pressure, sleep quality, metabolic and laboratory parameters, and treatment safety.

**Results:**

A reduction in 24‐h urinary protein excretion was observed in both treatment groups. The mean reduction was 52.45 ± 128.19 mg/day with melatonin and 28.69 ± 82.59 mg/day with placebo, yielding a between‐group difference of −23.76 mg/day (95% CI, −74.08 to 26.56; *p* = 0.92). Serum creatinine and estimated glomerular filtration rate remained largely stable during follow‐up. Compared with placebo, melatonin was associated with a significant reduction in total cholesterol. Sleep quality also showed a statistically significant difference in the change in PSQI score between groups, with mean changes of −0.18 ± 0.84 and −0.69 ± 1.06 in the melatonin and placebo groups, respectively (*p* = 0.047). No treatment‐related serious adverse events were observed.

**Conclusion:**

In adults with stage 3–4 CKD, 12 weeks of melatonin supplementation at 3 mg nightly did not demonstrate a clinically meaningful reduction in 24‐h urinary protein excretion compared with placebo. Although the point estimate favored melatonin, the confidence interval was compatible with both a modest benefit and no treatment effect. Melatonin was generally well tolerated and was associated with modest changes in selected metabolic and sleep‐related outcomes. Larger, multicenter trials with longer follow‐up and adequate statistical power are needed to determine whether melatonin has clinically relevant renal effects in specific CKD populations.

**Trial Registration:** This clinical trial was registered in the Iranian Registry of Clinical Trials (IRCT No. IRCT20160412027346N12)

## 1 Introduction

Chronic kidney disease (CKD) constitutes a major and growing health problem worldwide and is associated with substantial morbidity, mortality, and deterioration in quality of life [1, 2, 3, 4]. Its increasing prevalence has been attributed to the expanding burden of diabetes, hypertension, obesity, and population aging, and the global impact of CKD is expected to continue increasing in the coming decades [5]. The clinical course of CKD is influenced by multiple interacting mechanisms, including hemodynamic abnormalities, metabolic disturbances, neurohormonal changes, and progressive structural injury to the kidney [6, 7, 8]. Among the factors associated with CKD progression, persistent urinary protein loss has particular clinical importance. Proteinuria reflects disruption of the glomerular filtration barrier but may also participate directly in the progression of renal injury. Increased exposure of the renal tubules to filtered proteins can promote inflammatory and oxidative processes and contribute to tubulointerstitial damage and fibrosis [8, 9, 10, 11]. Consistent epidemiological evidence has linked higher levels of proteinuria with greater risks of kidney failure, cardiovascular complications, and mortality [12, 13, 14]. Reduction of proteinuria is therefore an important therapeutic objective, and established interventions that lower albuminuria, including renin–angiotensin system (RAS) blockade, have been associated with slower progression of CKD [1, 15, 16]. Proteinuria consequently serves both as a clinically relevant marker of renal injury and as an important outcome in CKD research. CKD is also accompanied by a broad range of systemic manifestations. In addition to cardiovascular, hematologic, metabolic, and mineral‐related complications, disturbances of sleep and circadian regulation are frequently reported, particularly among individuals with more advanced renal disease [3, 4, 17]. Altered sleep quality and circadian organization have been associated with autonomic and metabolic abnormalities as well as impaired vascular function in CKD populations [18, 19, 20]. These observations raise the possibility that interventions affecting circadian biology could have relevance beyond sleep‐related symptoms. Melatonin is an endogenous indoleamine that plays a central role in the regulation of circadian timing and the sleep–wake cycle [21]. Its biological activity, however, extends beyond chronobiological regulation. Experimental research has described antioxidant, anti‐inflammatory, anti‐apoptotic, and cytoprotective properties of melatonin [22, 23]. Because oxidative stress, inflammation, cellular injury, and dysregulation of renal signaling pathways contribute to CKD progression, melatonin has been proposed as a potentially relevant modulator of renal injury. Several lines of evidence provide biological support for this hypothesis. Experimental studies have suggested that melatonin may influence renal hemodynamics, preserve the integrity of podocytes and the glomerular filtration barrier, reduce intraglomerular pressure, and attenuate structural renal injury [24, 25, 26, 27, 28]. Human observational studies have likewise identified associations between altered nocturnal melatonin secretion and markers of renal injury, including albuminuria and impaired kidney function [29, 30, 31]. Dietary sodium intake may further modify these relationships, suggesting that circadian regulation, sodium sensitivity, and renal physiology may interact in complex ways [32]. Despite these findings, the translation of the experimental evidence into clinical benefit remains uncertain. Much of the available clinical literature involving melatonin in patients with CKD has concentrated on sleep or circadian disturbances rather than renal endpoints, and direct evidence regarding its effect on proteinuria in non‐dialysis CKD is limited [30]. Thus, the presence of a plausible biological mechanism does not establish that melatonin supplementation produces a clinically meaningful reduction in urinary protein excretion. The present study was designed to address this uncertainty in a controlled clinical setting. We conducted a randomized, double‐blind, placebo‐controlled trial to determine whether 12 weeks of oral melatonin supplementation affects 24‐h urinary protein excretion in adults with stage 3–4 CKD and persistent proteinuria. Changes in renal function, blood pressure, sleep quality, metabolic and laboratory parameters, and treatment safety were examined as secondary outcomes.

## 2 Methods

### 2.1 Study Design and Setting

This investigation was conducted as a prospective, randomized, double‐blind, placebo‐controlled clinical trial at Labbafinejad Hospital, a tertiary university‐affiliated referral center. Recruitment and follow‐up were performed from May 2024 through March 2025. The study was registered with the Iranian Registry of Clinical Trials (IRCT20160412027346N12) and followed the principles of the Declaration of Helsinki [33] and Good Clinical Practice guidelines.

The study protocol received approval from the Research Ethics Committee of the School of Pharmacy and Nursing & Midwifery, Shahid Beheshti University of Medical Sciences (IR.SBMU.PHARMACY.REC.1403.299). All participants provided written informed consent before enrollment and before any study‐related procedure was undertaken. Trial reporting followed the Consolidated Standards of Reporting Trials (CONSORT) recommendations [34].

### 2.2 Participants

Adults between 18 and 75 years of age were considered eligible when they had stage 3–4 CKD, defined by an estimated glomerular filtration rate (eGFR) of 15–59 mL/min/1.73 m^2^, stable renal function for at least 3 months, and no requirement for dialysis. Eligibility also required persistent proteinuria, defined as 24‐h urinary protein excretion of ≥ 300 mg/day and < 3000 mg/day, together with the ability and willingness to comply with the study procedures. Patients were not eligible if they had end‐stage kidney disease requiring hemodialysis or peritoneal dialysis, previous kidney transplantation, or an episode of acute kidney injury within the preceding 3 months. Additional exclusion criteria were uncontrolled hypertension (systolic blood pressure ≥ 180 mmHg or diastolic blood pressure ≥ 110 mmHg), poorly controlled diabetes (HbA1c ≥ 9%), active infection or inflammatory disease, autoimmune disorders, and clinically significant hepatic or gastrointestinal disease that could interfere with drug absorption or metabolism. A major cardiovascular event, including myocardial infarction or stroke, within the previous 3 months was also an exclusion criterion. Pregnancy or breastfeeding, a history of malignancy, and current or recent use of melatonin, sedative agents, antioxidant supplements, corticosteroids, or immunosuppressive drugs within 1 month before enrollment were additional exclusion criteria. Individuals with other conditions judged by the investigators to interfere with study participation or outcome assessment were also excluded.

### 2.3 Randomization, Masking, and Intervention

After completion of the baseline evaluation, participants who satisfied all eligibility requirements were assigned to treatment in equal proportions using a computer‐generated randomization sequence. Allocation was concealed through study‐specific treatment codes. Participants and investigators involved in data collection and outcome assessment remained unaware of treatment assignment throughout the intervention. Participants randomized to the active‐treatment arm received 3 mg of oral melatonin once daily at bedtime for 12 weeks. The comparator group received a placebo tablet designed to match the active formulation with respect to appearance, taste, smell, and packaging. Adherence was evaluated during scheduled follow‐up visits using pill counts. The selected dose and treatment schedule were based on previous clinical experience regarding the safety and use of melatonin [21, 29].

### 2.4 Sample Size Determination

The prespecified sample‐size calculation was based on the expected between‐group difference in the primary endpoint, namely the change in 24‐h urinary protein excretion. The calculation assumed a two‐sided *α* of 0.05, 80% statistical power, an anticipated mean difference of 12.66 units between treatment groups, and a standard deviation of 20 units. These assumptions corresponded to a moderate standardized effect size (Cohen's *d* ≈ 0.63) and indicated that 41 participants per group, or 82 participants in total, were required. Allowing for an anticipated dropout rate of approximately 5%, the recruitment target was increased to 88 participants. Seventy‐three participants ultimately completed the trial and were included in the final analysis. The original calculation was designed to detect a moderate treatment effect for the prespecified primary endpoint. Because the effect observed in the completed trial was substantially smaller, the available sample was less capable of detecting a small between‐group difference. A post hoc calculation based on the observed treatment effect yielded a Cohen's *d* of approximately 0.22 and an estimated statistical power of approximately 15%. These findings indicate that the study was underpowered to reliably identify small effects in the presence of the observed variability in proteinuria measurements.

### 2.5 Outcomes and Data Collection

The primary endpoint was the change in 24‐h urinary protein excretion from baseline to week 12. Prespecified secondary outcomes comprised urinary creatinine excretion, urine volume, renal function indices, blood pressure, sleep quality, lipid parameters, other laboratory measures, and treatment safety. Clinical and laboratory assessments were performed at baseline, before treatment initiation, and at week 12. Fasting blood samples and 24‐h urine specimens were collected at both time points. Laboratory assessments included serum creatinine, eGFR, serum electrolytes, liver enzymes, fasting blood glucose, lipid parameters, and complete blood count. All measurements were performed using standardized and validated laboratory procedures. Twenty‐4‐h urinary protein and urinary creatinine were determined using routine clinical laboratory methods in accordance with the manufacturer's analytical standards [27]. Baseline information included demographic characteristics, medical history, comorbidities, concomitant medications, height, weight, and body mass index (BMI). Blood pressure was measured after participants had remained seated for at least 5 min. Three consecutive readings were obtained with a calibrated sphygmomanometer, and their mean was used for analysis. Adherence was evaluated by pill counts and participant interviews at scheduled follow‐up visits. Safety surveillance was performed throughout the study, with adverse events documented at follow‐up assessments and at study completion. Events were classified according to their severity and their suspected relationship to the study medication. Concomitant medications and clinically relevant interventions were also recorded. In particular, use of renin–angiotensin–aldosterone system inhibitors and lipid‐lowering medications was documented at baseline. No protocol‐directed changes in background pharmacotherapy were required during the intervention. Nevertheless, residual confounding related to concomitant treatment cannot be completely excluded, particularly in view of the substantial burden of polypharmacy among patients with CKD.

### 2.6 Statistical Analysis

The statistical analysis followed a modified intention‐to‐treat (mITT) approach. Continuous variables were summarized as mean ± standard deviation (SD) when normally distributed and as median with interquartile range (IQR) when distributions were non‐normal. Categorical variables were expressed as counts and percentages. The principal efficacy analysis examined the between‐group difference in change in 24‐h urinary protein excretion from baseline to week 12. Secondary analyses considered changes in urinary creatinine, urine volume, serum creatinine, eGFR, blood pressure, sleep quality, lipid profile, laboratory parameters, and safety outcomes. No subgroup analyses were prespecified or conducted. Distributional characteristics were examined graphically using histograms. For between‐group comparisons, independent‐samples *t*‐tests were used for normally distributed continuous variables, whereas the Mann–Whitney *U* test was applied when the distribution was non‐normal. Paired *t*‐tests or Wilcoxon signed‐rank tests were used to assess within‐group changes. Categorical variables were compared using the chi‐square test, and Levene's test was used to evaluate homogeneity of variances. Changes across the two assessment points and treatment‐by‐time interactions were examined using Generalized Estimating Equations (GEE) with an exchangeable correlation structure. Treatment effects were expressed as between‐group differences in change from baseline together with 95% confidence intervals (CIs). *p* values were interpreted alongside effect estimates and their confidence intervals rather than as isolated indicators of evidence. All tests were two‐sided, with *α* = 0.05 specified a priori as the threshold for statistical significance. Analyses were performed using Stata version 17.0 (StataCorp LLC, College Station, TX, USA). Because the observed treatment effect on proteinuria was smaller than anticipated in the original sample‐size calculation, a post hoc power assessment was also performed. The observed standardized effect was approximately 0.22, corresponding to an estimated power of approximately 15%. Under these assumptions, approximately 326 participants per group would be required to achieve 80% power at a two‐sided *α* of 0.05. Such recruitment was not feasible within the context of the present single‐center trial. Accordingly, the findings concerning proteinuria should be regarded as preliminary and interpreted in light of the substantial variability of the outcome measure.

## 3 Results

### 3.1 Participant Flow and Treatment Exposure

A total of 88 individuals were screened for eligibility (Figure 1). Seven candidates were not enrolled because they either failed to satisfy the eligibility criteria (*n* = 5) or declined participation (*n* = 2). During follow‐up, eight participants discontinued the study because of headache, skin rash, nausea, or drowsiness. Consequently, 73 participants completed the 12‐week intervention and were available for the final analysis, including 37 individuals assigned to melatonin and 36 assigned to placebo. Overall adherence to the assigned treatment was high, and no serious adverse event attributable to either intervention was recorded.

**Figure 1:**
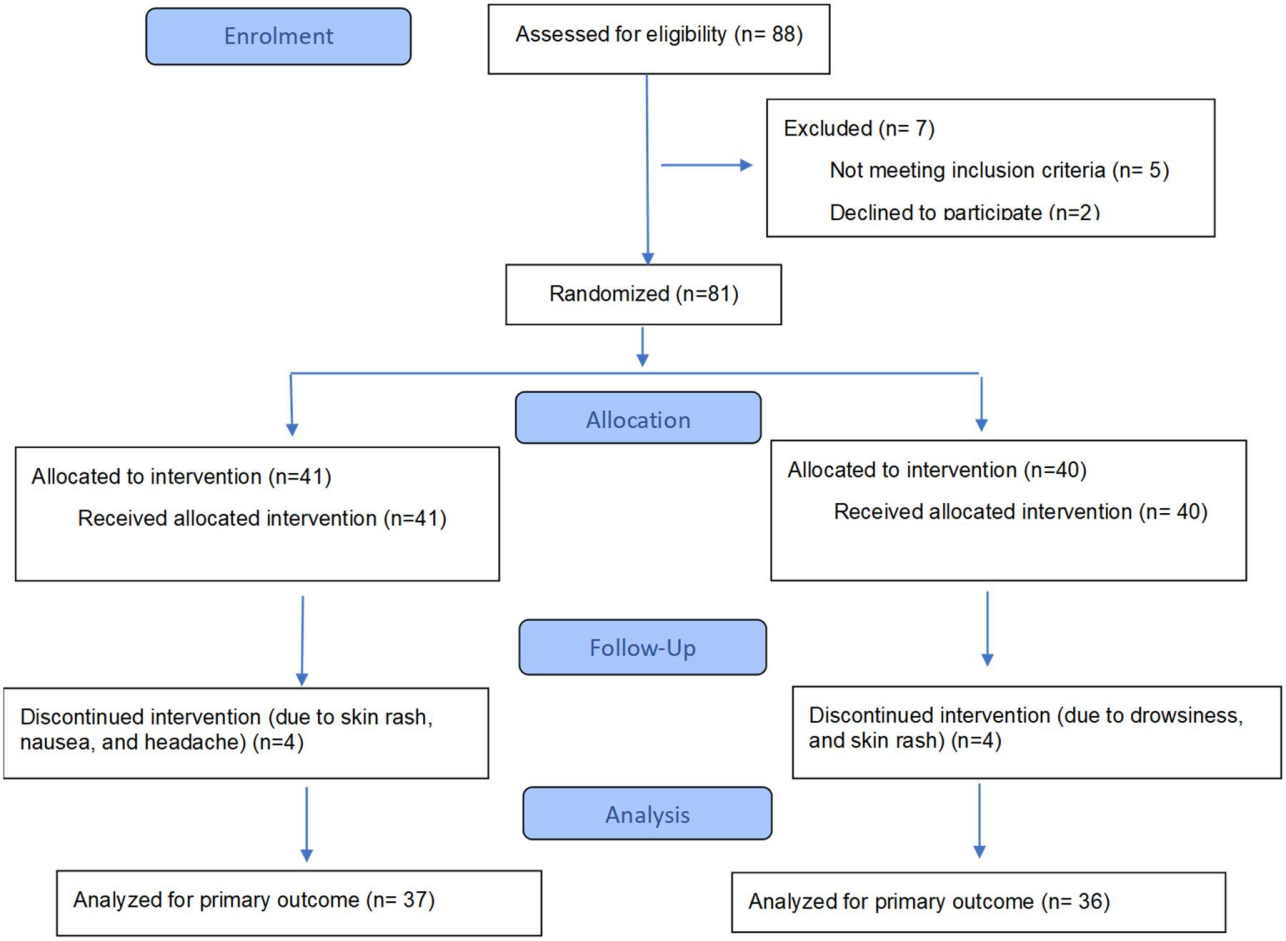
Study flowchart.

### 3.2 Baseline Characteristics

The demographic and clinical profiles of the two treatment groups are presented in Table 1. The groups were comparable at study entry across the principal demographic, clinical, comorbidity, adherence, and laboratory characteristics. Men accounted for 53.4% of the total study population, and the median age was 57 years (IQR, 42–67 years). Neither age (*p* = 0.13) nor sex distribution (*p* = 0.13) differed significantly between treatment groups. Treatment adherence was also similar between groups (*p* = 0.68), supporting adequate comparability following randomization.

**Table 1:** Baseline demographic and clinical characteristics of patients by intervention group.

Variables	Placebo (*n* = 36)	Melatonin (*n* = 37)	Total (*n* = 73)	*p*_value
General information				
Age (Years)	50.5 (39.5–65)	61 (49–69)	57 (42–67)	0.127
Gender				
Female	20 (55.56)	14 (37.84)	34 (46.58)	0.129
Male	16 (44.44)	23 (62.16)	39 (53.42)	0.129
Height (cm)	165.5 (160–175)	172 (163–180)	169 (162–178)	0.074
Weight (kg)	69 (63.5–79.5)	70 (65–75)	70 (64–78)	0.794
Body Mass Index (BMI, kg/m2)	24.65 (22.14–27.71)	23.45 (21.84–26.89)	24.21 (22.03–27.34)	0.440
Total intervention adherence (%)	91 (80–100)	90 (79–99)	91 (80–100)	0.675

*Note:* Data presented as median and interquartile range (Q1–Q3) and frequency and percentage.

### 3.3 Renal Outcomes and 24‐h Urinary Parameters

Baseline and week‐12 renal measurements and 24‐h urinary indices are provided in Table 2. None of the measured renal or urinary parameters showed a statistically significant difference between the treatment groups at baseline (all *p* > 0.05).

**Table 2:** Renal function and 24‐h urinary parameters of patients by intervention group during study.

Variables	Placebo (*n* = 36)	Pairwise comparison *p*‐value^b^	Melatonin (*n* = 37)	Pairwise comparison *p*‐value^b^	Between‐group comparison *p*‐value^c^
Urine volume (mL/24‐h)					
Before	1800 (1600–2250)	0.267	1700 (1500–2000)	0.910	0.132
After (3‐month intervention)	1800 (1650–2050)	0.267	1700 (1500–1900)	0.910	0.125
Difference^a^	16.67 ± 128.17	—	37.94 ± 335.25	—	0.415
Urine creatinine (mg/24‐h)					
Before	1295 (886.5–1553)	0.018*	1290 (950–1570)	0.007*	0.683
After (3‐month intervention)	1050 (900– 1513)	0.018*	1200 (900–1500)	0.007*	0.727
Difference^a^	−39.41 ± 96.02	—	−83.86 ± 183.85	—	0.531
Urine protein (mg/24‐h)					
Before	479 (170–1100)	0.059	500 (220–1670)	0.086	0.588
After (3‐month intervention)	495 (164–1088.5)	0.059	500 (200–1670)	0.086	0.634
Difference^a^	−28.69 ± 82.59	—	−52.45 ± 128.19	—	0.917
Serum creatinine (mg/dL)					
Before	1.7 (1.3–3.25)	< 0.001*	1.7 (1.4–2.35)	0.003*	0.933
After (3‐month intervention)	1.6 (1.3–3.0)	< 0.001*	1.6 (1.4–2.2)	0.003*	0.929
Difference^a^	−0.15 ± 0.26	—	−0.09 ± 0.17	—	0.327
BUN (mg/dL)					
Before	33 (21–51.5)	0.746	33 (23–45)	0.149	0.769
After (3‐month intervention)	33 (22–48)	0.746	35 (24–42)	0.149	0.584
Difference^a^	−1.00 ± 4.22	—	−0.94 ± 3.09	—	0.484

*Note:* Values described as median and interquartile range (Q1–Q3) or mean ± standard deviation,

^a^ Difference = After (3‐month intervention) value—baseline value,

^b^ Comparison of before intervention value with After (3‐month intervention) intervention value (paired comparison),

^c^ Comparison of the means between two groups of intervention.

* Statistically significant, *p*‐value < 0.05.

### 3.4 Primary Outcome: 24‐h Urinary Protein Excretion

The primary endpoint was defined as the change in 24‐h urinary protein excretion between baseline and week 12. A reduction in proteinuria occurred in both treatment arms during follow‐up. The mean decrease was 52.45 ± 128.19 mg/day among participants receiving melatonin and 28.69 ± 82.59 mg/day among those receiving placebo. The resulting between‐group difference in change was −23.76 mg/day (95% CI, −74.08 to 26.56; *p* = 0.92), corresponding to a Cohen's *d* of 0.22. Thus, although the point estimate numerically favored melatonin, the confidence interval was compatible with both a modest reduction and the absence of a treatment effect. The observed effect was substantially smaller than the moderate effect assumed in the original sample‐size calculation. A post hoc analysis based on the observed effect size estimated statistical power at approximately 15%, compared with the prespecified target of 80%. Accordingly, the study had limited ability to distinguish a small treatment effect from random variation in urinary protein measurements.

### 3.5 Secondary Renal Outcomes

Urinary creatinine excretion declined during follow‐up in both groups. The numerical reduction was greater in the melatonin arm (−83.86 ± 183.85) than in the placebo arm (−39.41 ± 96.02); however, this difference between groups was not statistically significant (*p* = 0.53). No significant between‐group differences were identified for urine volume, serum creatinine, eGFR, or the other assessed renal function measures (Table 2).

### 3.6 Sleep Quality and Blood Pressure

Results for PSQI and blood pressure are summarized in Table 3. Baseline PSQI scores and systolic blood pressure were similar between groups (*p* > 0.05). During the 12‐week intervention, PSQI scores declined in both arms. The within‐group reduction reached statistical significance in the placebo group (*p* < 0.001), whereas the corresponding change in the melatonin group was not statistically significant (*p* = 0.267). The mean change in PSQI score was −0.18 ± 0.84 in the melatonin group and −0.69 ± 1.06 in the placebo group. The difference in change between the two groups was statistically significant (*p* = 0.047). The proportion of participants classified as having poor sleep quality also decreased modestly during follow‐up in both treatment arms.

**Table 3:** Sleep quality and blood pressure assessment of patients by intervention group during study.

Variables	Placebo (*n* = 36)	Pairwise comparison *p*‐value^b^	Melatonin (*n* = 37)	Pairwise comparison *p*‐value^b^	Between‐group comparison *p*‐value^c^
Pittsburgh Sleep Quality Index (PSQI score)					
Before	5.5 (2–12.5)	< 0.001*	7 (3–12)	0.267	0.803
After (3‐month intervention)	5 (2–11)	< 0.001*	7 (2–12)	0.267	0.591
Difference^a^	−0.69 ± 1.06	—	−0.18 ± 0.84	—	0.047*
Pittsburgh Sleep Quality Index (PSQI category)					
Before		—		—	0.845
Poor	11 (30.56)	—	13 (35.14)	—	0.845
Moderate	8 (22.22)	—	9 (24.32)	—	0.845
Good	17 (47.22)	—	15 (40.54)	—	0.845
After (3‐month intervention)					
Poor	10 (27.78)	0.563	11 (29.73)	0.157	0.836
Moderate	9 (25.00)	0.563	11 (29.73)	0.157	0.836
Good	17 (47.22)	0.563	15 (40.54)	0.157	0.836
Difference^a^	—	—	—	—	—
Systolic blood pressure (mmHg)					
Before	130 (120–140)	0.096	130 (120–135)	0.012*	0.924
After (3‐month intervention)	130 (120–140)	0.096	130 (120–140)	0.012*	0.841
Difference^a^	2.36 ± 7.69	—	1.94 ± 4.60	—	0.768
Diastolic blood pressure (mmHg)					
Before	90 (80–90)	0.669	90 (80–90)	0.541	0.680
After (3‐month intervention)	90 (80–90)	0.669	90 (80–90)	0.541	0.311
Difference^a^	0.55 ± 4.74	—	−0.13 ± 3.99	—	0.514

*Note:* Values described as median and interquartile range (Q1–Q3) or mean ± standard deviation.

^a^ Difference = After (3‐month intervention) value—baseline value.

^b^ Comparison of before‐intervention value with after‐intervention value (paired comparison).

^c^ Comparison of the means between two intervention groups.

* Statistically significant, *p*‐value < 0.05.

Systolic blood pressure rose by 1.94 ± 4.60 mmHg in the melatonin group (*p* = 0.012), whereas the change in the placebo group was not statistically significant (*p* = 0.096). Importantly, the corresponding between‐group comparison did not demonstrate a significant difference (*p* = 0.768).

### 3.7 Metabolic Outcomes

The lipid and glycemic findings are presented in Table 4. Baseline lipid concentrations and fasting blood glucose were comparable between the treatment groups (*p* > 0.05). During follow‐up, total cholesterol decreased by 1.02 ± 2.96 in the melatonin group (*p* < 0.05), whereas no significant within‐group change was observed with placebo. The between‐group comparison indicated a statistically significant difference favoring melatonin (*p* = 0.029). In contrast, LDL cholesterol increased within the melatonin group (*p* = 0.020), while the change in the placebo group did not reach statistical significance (*p* = 0.062). Despite these within‐group findings, the change in LDL cholesterol did not differ significantly between treatment groups (*p* = 0.675). HDL cholesterol increased significantly in the placebo group (*p* = 0.030) but remained statistically unchanged in the melatonin group. The difference between groups was not significant (*p* = 0.173). Neither triglyceride concentrations nor fasting blood glucose demonstrated significant changes within or between treatment groups during the intervention (all *p* > 0.05).

**Table 4:** Lipid and glycemic profiles of patients by intervention group during study.

Variables	Placebo (*n* = 36)	Pairwise comparison *p*‐value^b^	Melatonin (*n* = 37)	Pairwise comparison *p*‐value^b^	Between‐group comparison *p*‐value^c^
Total cholesterol (mg/dL)					
Before	152 (124.5–184.5)	0.426	153 (135–179)	0.042*	0.896
After (3‐month intervention)	152 (125–184.5)	0.426	153 (135–175)	0.042*	0.781
Difference^a^	0.19 ± 1.45	—	−1.02 ± 2.96	—	0.029*
LDL (mg/dL)					
Before	79.5 (57.5–102)	0.062	81 (64–100)	0.020*	0.514
After (3‐month intervention)	80.5 (57.5–102.5)	0.062	84 (65–100)	0.020*	0.466
Difference^a^	0.50 ± 1.79	—	0.48 ± 1.32	—	0.675
HDL (mg/dL)					
Before	40.5 (35–49.5)	0.030*	40 (36–49)	0.560	0.929
After (3‐month intervention)	40.5 (35.5–50.5)	0.030*	40 (37–49)	0.560	0.829
Difference^a^	0.97 ± 3.35	—	0.02 ± 1.04	—	0.173
Triglyceride (mg/dL)					
Before	140.5 (94–181)	0.522	130 (107–188)	0.682	0.808
After (3‐month intervention)	141.5 (94–180.5)	0.522	130 (107–188)	0.682	0.757
Difference^a^	−0.89 ± 6.35	—	−0.02 ± 1.32	—	0.451
Fasting blood sugar (FBS, mg/dL)					
Before	95.5 (83–107.5)	0.197	97 (90–110)	0.180	0.304
After (3‐month intervention)	95.5 (83.5–105)	0.197	97 (90–110)	0.180	0.291
Difference^a^	0.08 ± 2.03	—	0.27 ± 1.23	—	0.970

*Note:* Values described as median and interquartile range (Q1–Q3) or mean ± standard deviation.

^a^ Difference = After (3‐month intervention) value—baseline value.

^b^ Comparison of before intervention value with after (3‐month intervention) intervention value (paired comparison).

^c^ Comparison of the means between two groups of intervention.

* Statistically significant, *p*‐value < 0.05.

### 3.8 Laboratory Parameters

The results for hematologic parameters, liver enzymes, and serum electrolytes are presented in Table 5. At baseline, hematologic parameters, liver enzymes, and serum electrolyte concentrations were comparable between the melatonin and placebo groups. During the 3‐month intervention, AST levels increased significantly in the placebo group (*p* = 0.034), whereas no significant change was observed in the melatonin group (*p* = 0.915). However, the between‐group difference in AST changes was not statistically significant (*p* = 0.097). Serum potassium levels decreased significantly in the melatonin group (*p* < 0.001), while no significant change was observed in the placebo group (*p* = 0.230). Nevertheless, the between‐group comparison of changes in serum potassium did not reach statistical significance (*p* = 0.073). No significant changes were observed in ALT or serum sodium levels in either group.

**Table 5:** Blood factors, liver enzymes and serum electrolytes of patients by intervention group during study.

Variables	Placebo (*n* = 36)	Pairwise comparison *p*‐value^b^	Melatonin (*n* = 37)	Pairwise comparison *p*‐value^b^	Between‐group comparison *p*‐value^c^
WBC (10^9^/L)					
Before	6.47 (5.60–7.45)	0.680	6.5 (5.4–7.6)	0.338	0.864
After (3‐month intervention)	6.47 (5.80–7.45)	0.680	6.5 (5.4–7.5)	0.338	0.825
Difference^a^	0.01 ± 0.13	—	−0.004 ± 0.14	—	0.845
Platelets (10^9^/L)					
Before	225 (188.5–274.5)	0.674	202 (178–267)	0.428	0.493
After (3‐month intervention)	225 (186–274.5)	0.674	202 (181–267)	0.428	0.483
Difference^a^	−0.11 ± 2.70	—	−0.11 ± 7.56	—	0.783
Hemoglobin (g/dL)					
Before	13 (12–14)	0.114	13.1 (12–14.6)	0.468	0.434
After (3‐month intervention)	13 (12–14)	0.114	13 (12–14.5)	0.468	0.424
Difference^a^	−0.04 ± 0.24	—	−0.02 ± 0.35	—	0.786
ALT (U/L)					
Before	20 (15–29)	0.688	20 (15–25)	0.313	0.748
After (3‐month intervention)	20 (15.5–28)	0.688	20 (15–25)	0.313	0.744
Difference^a^	0.08 ± 0.99	—	0.13 ± 1.10	—	0.669
AST (U/L)					
Before	19.5 (15.5–24.5)	0.034*	21 (18–24)	0.915	0.439
After (3‐month intervention)	20 (16–24.5)	0.034*	21 (18–23)	0.915	0.542
Difference^a^	0.41 ± 1.18	—	−0.08 ± 0.86	—	0.097
Serum sodium (Na, mEq/L)					
Before	139.5 (137.5–141)	0.193	140 (138–141)	0.087	0.713
After (3‐month intervention)	140 (138–141.5)	0.193	140 (138–141)	0.087	0.885
Difference^a^	0.36 ± 1.60	—	0.21 ± 1.20	—	0.578
Serum potassium (K, mmol/L)					
Before	4.3 (4–4.6)	0.230	4.4 (4.2–4.7)	< 0.001*	0.101
After (3‐month intervention)	4.3 (4–4.6)	0.230	4.3 (4.2–4.5)	< 0.001*	0.185
Difference^a^	−0.02 ± 0.13	—	−0.07 ± 0.12	—	0.073

*Note:* Values described as median and interquartile range (Q1–Q3) or mean ± standard deviation.

^a^ Difference = After (3‐month intervention) value—baseline value.

^b^ Comparison of before intervention value with after (3‐month intervention) value (paired comparison).

^c^ Comparison of the means between two groups of intervention.

* Statistically significant, *p*‐value < 0.05.

### 3.9 Longitudinal Analysis

The longitudinal trajectories of the major laboratory and clinical outcomes are illustrated in Figures 2, 3, 4, 5. GEE analyses identified significant changes over time in total cholesterol and LDL cholesterol within the melatonin group (both *p* < 0.05); however, the treatment‐by‐time interactions for these variables were not statistically significant (Figure 2). For systolic blood pressure, the temporal change within the melatonin group was significant (*p* = 0.005), and a significant treatment‐by‐time interaction was also detected (*p* = 0.013), indicating that the pattern of change over the study period differed between the treatment arms (Figure 3). For serum creatinine, both the effect of time and the treatment‐by‐time interaction were statistically significant (both *p* < 0.05). BUN demonstrated a significant temporal change within the melatonin group (*p* = 0.016), accompanied by a significant treatment‐by‐time interaction (*p* = 0.020). Serum potassium similarly showed a significant within‐group temporal change in the melatonin arm (*p* < 0.001) and a significant treatment‐by‐time interaction (*p* = 0.003) (Figure 4). No statistically significant temporal changes or treatment‐by‐time interactions were detected for hematologic indices, fasting blood glucose, liver enzymes, or serum sodium (Figure 5).

**Figure 2:**
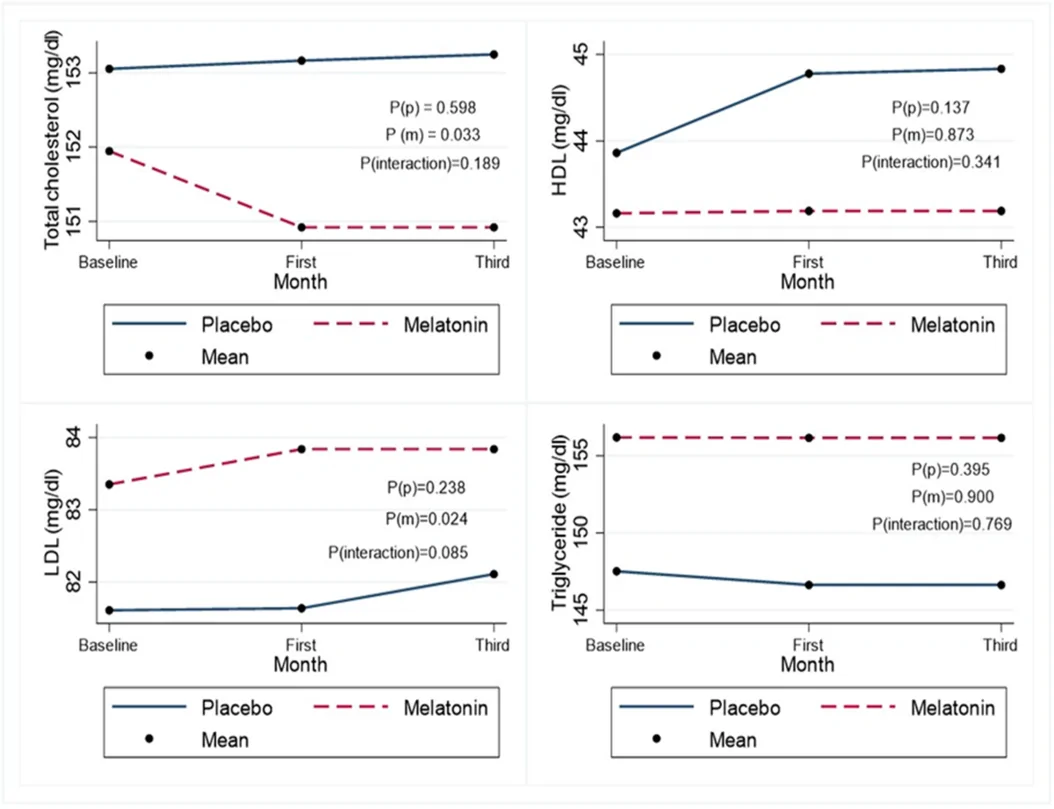
Longitudinal changes in lipid profile (Total cholesterol, HDL, LDL, Triglyceride) over the 12‐week intervention period.

**Figure 3:**
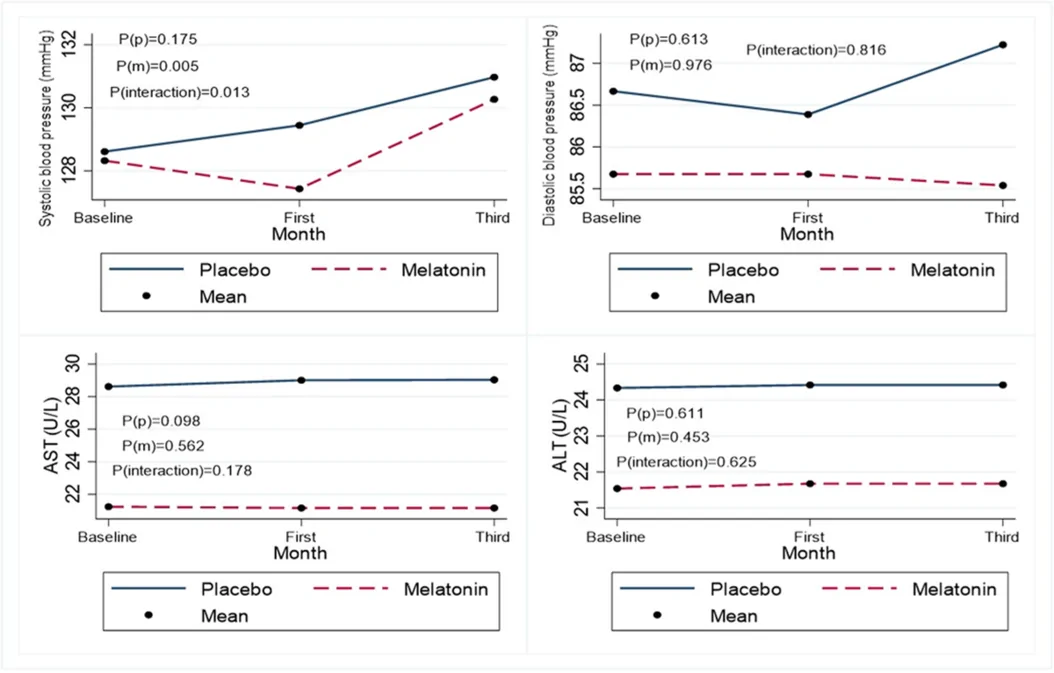
Longitudinal changes in systolic and diastolic blood pressure and liver enzymes over the 12‐week intervention period.

**Figure 4:**
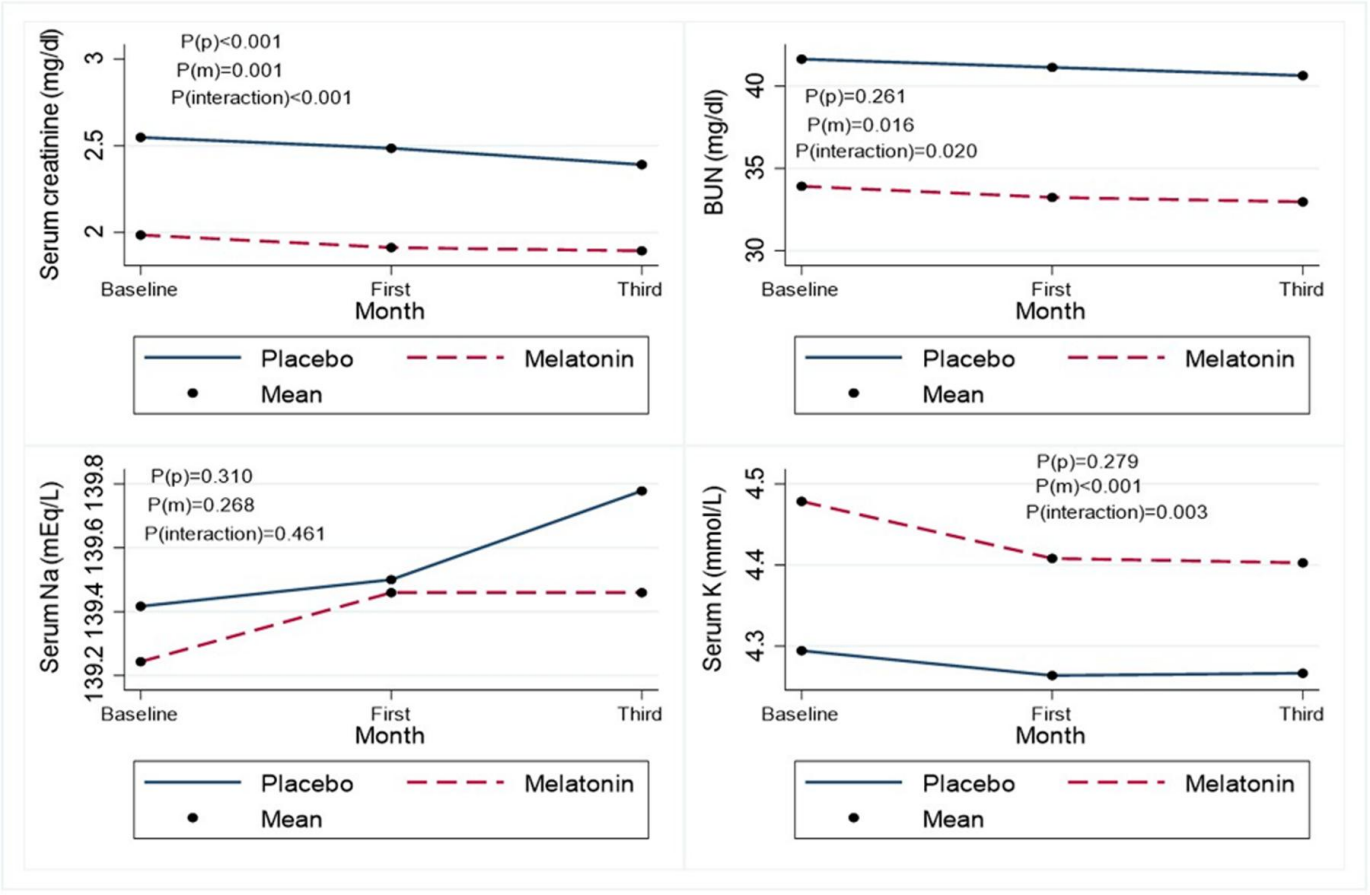
Longitudinal changes in serum creatinine, blood urea nitrogen (BUN), serum sodium, and serum potassium over the 12‐week intervention period.

**Figure 5:**
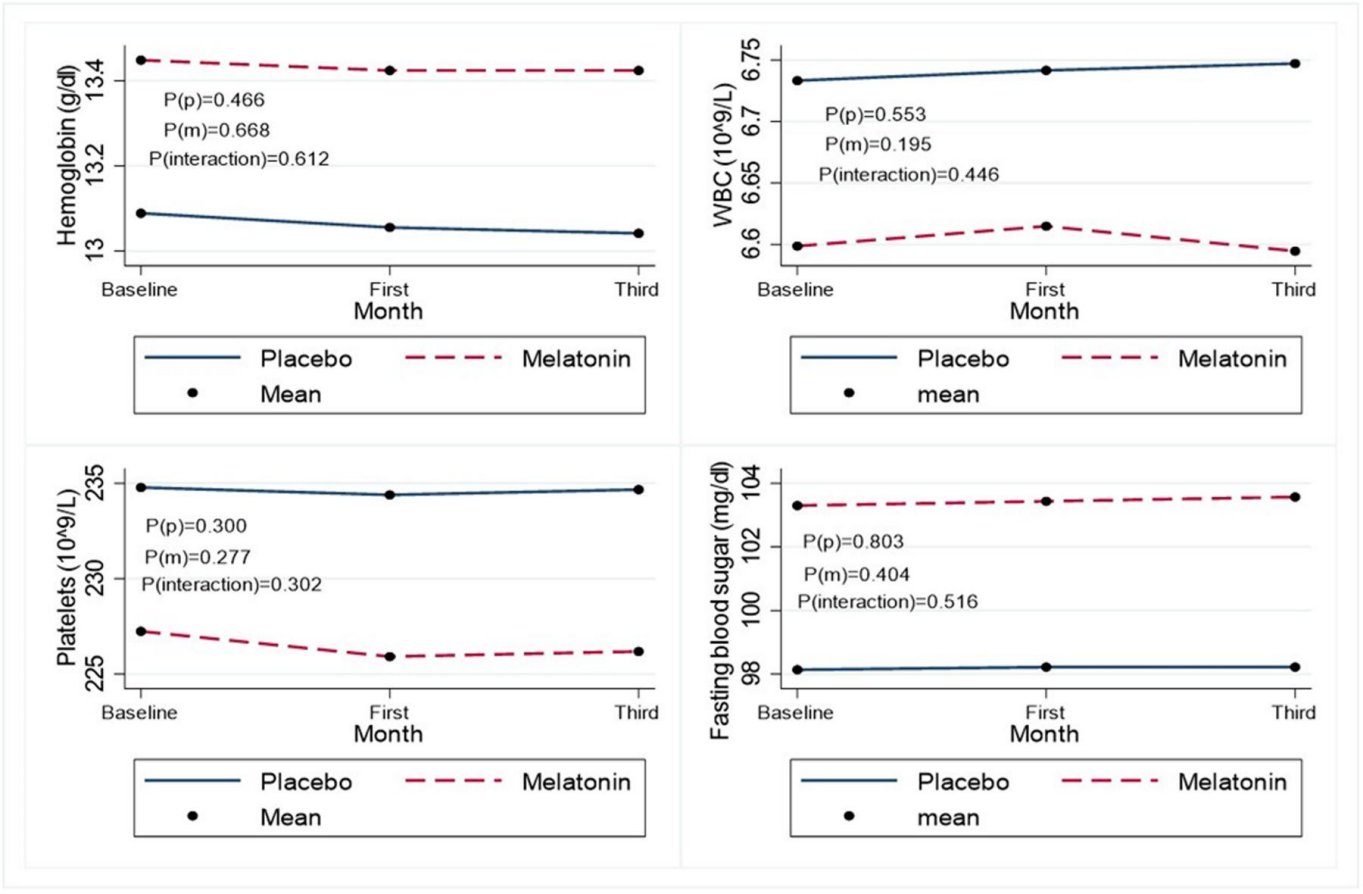
Longitudinal changes in blood indices and fasting blood sugar (FBS) over the 12‐week intervention period.

### 3.10 Safety Outcomes

Both treatment regimens were well tolerated over the 12‐week study period. No serious adverse event considered related to treatment was reported. Mild and transient complaints, including sleepiness, headache, minor skin reactions, and gastrointestinal discomfort, were reported infrequently and occurred at comparable frequencies in the two groups. Overall, the safety findings did not identify a clinically important treatment‐related safety signal at the evaluated melatonin dose.

## 4 Discussion

In this randomized, double‐blind, placebo‐controlled trial, we investigated whether 12 weeks of nightly melatonin supplementation could modify 24‐h urinary protein excretion in adults with stage 3–4 chronic kidney disease (CKD). The primary analysis did not demonstrate a statistically significant difference between melatonin and placebo. Although the numerical estimate of the treatment effect favored melatonin, the magnitude of the difference was small and associated with considerable uncertainty. The adjusted between‐group difference in change from baseline was −23.76 mg/day, with a 95% confidence interval extending from −74.08 to 26.56 mg/day. Thus, the results do not establish a clinically meaningful antiproteinuric effect of 3 mg melatonin administered nightly for 12 weeks.

### 4.1 Relationship to Previous Human Evidence

The biological relationship between melatonin and renal injury has been investigated in several observational studies. Alterations in endogenous melatonin secretion have been linked to markers of glomerular damage and protein loss in patients with CKD. Ishigaki et al. reported that lower urinary concentrations of 6‐sulfatoxymelatonin (aMT6s), commonly used as an indicator of nocturnal melatonin production, were associated with greater proteinuria and albuminuria and with increased urinary angiotensinogen. Their findings raise the possibility that abnormalities in melatonin secretion may be related to activation of the intrarenal renin–angiotensin system (RAS) and impaired regulation of the glomerular filtration barrier [27]. The interaction between melatonin and dietary sodium may also be relevant. Ohashi et al. observed a stronger inverse relationship between melatonin levels and proteinuria among individuals with higher salt intake, suggesting that circadian regulation, sodium sensitivity, and renal injury may not operate independently in CKD [32]. Other investigations have described progressive disturbances in melatonin secretion with worsening CKD, including attenuation of the nocturnal peak, reduced rhythm amplitude, and delayed timing of secretion. Such abnormalities could potentially influence hemodynamic and neurohormonal pathways involved in renal injury and proteinuria [35]. These observational findings, however, should not be interpreted as evidence that restoration of melatonin signaling necessarily produces a measurable reduction in proteinuria. An association between endogenous melatonin patterns and renal disease may reflect the broader physiological disturbances accompanying CKD rather than a directly modifiable causal pathway. The neutral primary outcome of the present trial therefore provides an important clinical counterpoint to the associations reported in observational studies.

### 4.2 Evidence From Preclinical Models

Experimental studies provide several plausible mechanisms through which melatonin could exert renoprotective effects. In the 5/6 nephrectomy model, melatonin administration was associated with lower proteinuria, preservation of glomerular architecture, and attenuation of structural renal injury [36]. Findings from salt‐sensitive and high‐salt hypertension models have similarly suggested beneficial effects on podocyte integrity and albuminuria, accompanied by reductions in oxidative damage within the glomerulus [37]. In models of obstructive nephropathy, melatonin has also been reported to attenuate tubular and interstitial injury and to limit pathological renal remodeling [38]. Taken together, these experimental observations suggest that melatonin can influence several pathways implicated in kidney injury, including oxidative stress, inflammatory signaling, disruption of the glomerular filtration barrier, and fibrotic remodeling. Nevertheless, mechanistic activity observed in experimental models does not necessarily translate into a clinically detectable effect in patients with established CKD. Differences in disease severity, treatment exposure, duration, concomitant medications, and the complexity of human CKD may all contribute to this discrepancy.

### 4.3 Interpretation in the Context of Clinical Trials

Compared with the experimental literature, randomized clinical evidence concerning the renal effects of melatonin remains limited. Previous clinical studies involving patients with diabetic CKD have primarily examined metabolic or inflammatory outcomes and have generally not demonstrated clear improvements in renal endpoints [39]. In addition, much of the clinical literature on melatonin in CKD has focused on patients receiving dialysis or on sleep and circadian disturbances rather than on proteinuria as a prespecified renal outcome. The present trial therefore adds randomized evidence addressing a more specific question whether melatonin supplementation can reduce 24‐h urinary protein excretion in patients with stage 3–4 non‐dialysis CKD. The absence of a significant treatment effect suggests that the biological mechanisms proposed in observational and preclinical research may not be sufficient, at least at the dose and duration examined here, to produce a clinically meaningful reduction in proteinuria.

### 4.4 Clinical Relevance and Treatment Considerations

From a clinical perspective, the current findings do not support the use of short‐term melatonin supplementation as an established treatment for proteinuria in stage 3–4 CKD. Proteinuria declined during follow‐up in both study groups, but the difference in the magnitude of change between melatonin and placebo was limited. At the same time, the intervention was generally well tolerated, and no serious adverse event attributable to treatment was identified. This safety profile is relevant in CKD, where patients frequently receive multiple medications and may consequently be vulnerable to treatment‐related adverse effects. Several secondary findings merit consideration but should not be interpreted as definitive evidence of additional therapeutic effects. Total cholesterol decreased significantly among participants receiving melatonin, with a significant difference between groups. Because the absolute magnitude of this change was modest, its clinical importance remains uncertain. Conversely, LDL cholesterol increased within the melatonin group, although the corresponding between‐group comparison was not significant. The small absolute change may represent biological or measurement variability and could also have been influenced by concomitant lipid‐lowering treatment or other changes during follow‐up. A similar cautious interpretation applies to the observed changes in systolic blood pressure and serum potassium. Systolic blood pressure increased modestly during follow‐up in the melatonin group, but the magnitude of the change was unlikely to represent a clinically important effect and the between‐group comparison was not significant. Serum potassium decreased significantly within the melatonin group, although the between‐group comparison did not reach statistical significance. The reported values remained within the physiological range and no potassium‐related adverse events were observed. These secondary observations therefore require confirmation before any clinical implications can be assigned to them. The dose and treatment duration may also have influenced the primary outcome. We selected 3 mg of melatonin administered nightly for 12 weeks based on its established clinical use and favorable safety profile. However, urinary protein excretion can show substantial biological variability, and changes that are relevant to long‐term CKD progression may not become apparent over a relatively short treatment period. The relatively small numerical reduction observed in the melatonin group also suggests that, if melatonin has an antiproteinuric effect, its magnitude may be modest. Consequently, a longer intervention period, a larger sample, or a different dosing strategy may be required to adequately evaluate this possibility. Future trials should therefore consider follow‐up periods of at least 6 months, repeated measurements of proteinuria, and adequate statistical power to detect small between‐group differences. Evaluation of alternative melatonin doses and formulations may also help determine whether renal effects are dependent on the extent or duration of melatonin exposure.

### 4.5 Strengths and Limitations

Several methodological features strengthen the present study. The randomized, double‐blind, placebo‐controlled design reduces the likelihood of systematic differences between treatment groups and minimizes expectation‐related bias. In addition, 24‐h urinary protein excretion was selected as a clinically relevant renal outcome, participants were followed prospectively under a consistent protocol, and safety outcomes were actively monitored. Treatment adherence was also favorable, and the study was conducted and reported according to established standards for clinical trials. The limitations, however, should be considered when interpreting the neutral primary outcome. Most importantly, the relatively small sample size substantially limited the ability to detect a small treatment effect. Based on the observed between‐group effect size, the post hoc statistical power was approximately 15%. Therefore, the absence of statistical significance should not be interpreted as definitive evidence that melatonin has no effect on proteinuria. Rather, the data are compatible with a small treatment effect that could not be distinguished reliably from the substantial variability in urinary protein measurements. The 12‐week intervention period represents a second important limitation. Proteinuria may fluctuate over time, and a longer period may be necessary to establish whether an intervention produces a sustained renal effect or influences subsequent CKD progression. In addition, the single‐center setting may restrict the generalizability of the findings to other CKD populations. Another limitation was the absence of direct measurement of serum or urinary melatonin concentrations. Consequently, we could not determine interindividual differences in melatonin exposure, pharmacokinetic variability, or the relationship between achieved melatonin concentrations and clinical response. Although treatment adherence was assessed, pill‐based adherence measures cannot establish the extent of biological exposure. The assessment of sleep quality also relied on the Pittsburgh Sleep Quality Index (PSQI), which is a validated but subjective measure. Incorporation of objective approaches, such as actigraphy or polysomnography, could provide a more comprehensive assessment of sleep and circadian outcomes in future studies. Finally, several potentially important determinants of treatment response were not fully characterized. Dietary sodium intake, pre‐existing circadian abnormalities, and changes in concomitant RAS‐blocking or sodium–glucose cotransporter 2 (SGLT2) inhibitor therapy could have affected urinary protein excretion during follow‐up. Moreover, because individuals with uncontrolled diabetes or hypertension were excluded, the findings may not be directly applicable to CKD populations with more advanced metabolic or cardiovascular risk.

### 4.6 Future Research

The current findings highlight several priorities for subsequent investigation. Larger, multicenter randomized trials with longer follow‐up are needed to determine whether sustained melatonin exposure can influence proteinuria or the trajectory of renal function. Such studies should be sufficiently powered to detect relatively small treatment effects, given the modest effect observed in the present trial. Dose‐ranging studies and comparisons of immediate‐release and sustained‐release formulations could help establish the optimal exposure for patients with CKD. Measurement of circulating or urinary melatonin concentrations would also allow pharmacokinetic characteristics and treatment exposure to be incorporated into efficacy analyses. Because CKD represents a heterogeneous group of disorders, future investigations may benefit from prespecified subgroup analyses. Patients with greater baseline proteinuria, salt‐sensitive disease, or marked circadian disruption may represent populations in whom a biological response to melatonin is more likely to be detectable [40, 41, 42]. Such analyses should be prospectively planned and adequately powered rather than interpreted from small exploratory subgroups. Another important question is whether melatonin could provide an effect when added to established renoprotective therapy rather than being evaluated as an isolated intervention. Studies combining melatonin with RAS blockade or SGLT2 inhibitors may clarify whether its biological actions complement existing antiproteinuric strategies or provide additional benefit through mechanisms not adequately addressed by current therapies. Finally, mechanistic clinical studies incorporating podocyte‐related biomarkers, renal imaging, omics‐based analyses, and objective assessment of circadian rhythms could help determine whether specific biological phenotypes are associated with a response to melatonin. Such approaches may be particularly useful for explaining why promising experimental mechanisms have not yet translated into a clearly demonstrated antiproteinuric effect in clinical trials.

## 5 Conclusion

Among adults with stage 3–4 chronic kidney disease and persistent proteinuria, 12 weeks of nightly melatonin supplementation at a dose of 3 mg did not demonstrate a clinically meaningful reduction in 24‐h urinary protein excretion compared with placebo. Although the estimated treatment effect favored melatonin, its magnitude was small and associated with substantial uncertainty, while the limited sample size restricted the ability to detect modest effects. The intervention was generally well tolerated, with some changes observed in sleep‐related and metabolic measures that require further confirmation. These findings do not establish melatonin as an antiproteinuric therapy in CKD. However, they support further adequately powered, multicenter trials with longer follow‐up and assessment of melatonin exposure and circadian characteristics to determine whether specific patient populations or alternative treatment strategies may derive renal benefit.

## Author Contributions

**Shadi Ziaie:** conceptualization, methodology, supervision, project administration, validation. **Behnam Mahdavi:** project administration, resources, validation. **Fatemeh Pourrezagholi:** conceptualization, supervision, project administration, resources. **Aylar Nadiri Niri:** resources, investigation, project administration, visualization. **Mohammad Abbasinazari:** resources, supervision, validation. **Roja Asadpour:** formal analysis, writing – original draft, writing – review and editing, investigation, funding acquisition, software, data curation.

## Funding

The authors have nothing to report.

## Ethics Statement

This study was conducted in accordance with the ethical principles of the Declaration of Helsinki and Good Clinical Practice guidelines. Ethical approval was obtained from the Research Ethics Committee of the School of Pharmacy and Nursing & Midwifery, Shahid Beheshti University of Medical Sciences (Approval No. IR.SBMU.PHARMACY.REC.1403.299).

## Consent

Written informed consent was obtained from all participants prior to enrollment and before the initiation of any study‐related procedures.

## Conflicts of Interest

The authors declare no conflicts of interest.

## Author Declaration and Guarantor Statement

All authors have read and approved the final version of the manuscript. Roja Asadpour, the corresponding author and manuscript guarantor, had full access to all study data and takes complete responsibility for the integrity of the data and the accuracy of the data analysis.

## Transparency Statement

Roja Asadpour, the corresponding author and manuscript guarantor, affirms that this manuscript is an honest, accurate, and transparent account of the study being reported; that no important aspects of the study have been omitted; and that any discrepancies from the study as originally planned and registered (IRCT20160412027346N12) have been fully disclosed and explained in the manuscript.

## Data Availability

The data sets generated and analyzed during the current study are not publicly available because they contain information that could compromise the privacy of research participants. De‐identified data supporting the findings of this study are available from the corresponding author upon reasonable request, subject to institutional approval and applicable ethical and regulatory requirements.
